# Comparison of Various Theoretical Measures of Aromaticity within Monosubstituted Benzene

**DOI:** 10.3390/molecules29102260

**Published:** 2024-05-11

**Authors:** Caleb K. Swain, Steve Scheiner

**Affiliations:** Department of Chemistry and Biochemistry, Utah State University, Logan, UT 84322-0300, USA

**Keywords:** substituent effect, AIM, NICS, HOMA, aromatic stabilization energy

## Abstract

The effects of monosubstitution on the aromaticity of benzene are assessed using a number of different quantitative schemes. The ability of the mobile π-electrons to respond to an external magnetic field is evaluated using several variants of the NICS scheme which calculate the shielding of points along the axis perpendicular to the molecule. Another class of measures is related to the drive toward the uniformity of C-C bond lengths and strengths. Several energetic quantities are devised to approximate an aromatic stabilization energy and the tendency of the molecule to maintain planarity. There is a lack of consistency in that the various measures of aromaticity lead to differing conclusions as to the effects of substituents on the aromaticity of the ring.

## 1. Introduction

The concept of aromaticity [1,2] has been one of the most foundational and far-reaching principles in all of chemistry. Originally proposed to help explain some of the properties of the simple benzene ring, aromaticity has extended its tentacles to reach into many areas of organic, biological, and physical chemistry, and the ideas have propagated to systems quite different than the original target benzene [3,4,5]. Its ideas have been used to explain rings of various sizes, rings that contain heteroatoms, and polycyclic systems from naphthalene up to graphene.

At its heart, aromaticity occurs when the alternating single/double bonding pattern within the Kekule structure of a phenyl ring is merged into a set of fully equivalent bonds, each of which lies roughly halfway between C-C and C=C. That is, all bond lengths in benzene are equal, longer, and weaker than a classic C=C bond as in ethylene, but shorter and stronger than the C-C bond of ethane. This bond equalization is thought to stabilize the system, relative to the Kekule structure, with its bond-alternating character. The molecular orbital pattern of the π-system contains one orbital of lowest energy that has equal contributions from all of the C p_z_ orbitals; the higher-lying π-orbitals then occur in degenerate pairs. The 4n + 2 rule has emerged as a guiding principle as to the number of π-electrons that fill these orbitals. Because of the fully delocalized nature of these orbitals, the electrons can easily respond to an externally imposed magnetic field in such a way as to produce an internal field that opposes the external one within the confines of the ring [3,6]. The resulting magnetic lines of force deshield the NMR signals of nuclei that lie outside the perimeter of an aromatic system

While there is no question concerning the aromaticity of benzene, naphthalene, pyridine, and their various derivatives, one issue of interest concerns a quantitative measure of the level of aromaticity, and how this parameter is affected by substituents, electronic excitation, and so forth. For example, would an excited triplet or singlet state of a system of this sort make the molecule more or less aromatic? Does the second ring of naphthalene make this molecule more or less aromatic than benzene with its single ring? What is the effect of a highly extended system as in graphene? Or how might the replacement of one H atom of benzene by, say, Cl, affect its aromaticity?

Despite its pivotal station in chemistry, a true quantitative definition remains elusive [2,7]. Nonetheless, there have been numerous attempts to arrive at such a definition [4,8,9,10,11]. One set of measures derive from the ability of the loosely held π-electrons to respond to an external magnetic field [12,13,14]. The most popular of these, the nucleus-independent chemical shift (NICS) procedure [15], evaluates shielding at certain points in space, usually lying along an axis that is perpendicular to the molecular plane and passing through its center. There have also been other means of considering magnetic effects as they relate to aromaticity [16,17,18]. Another family of quantifications are based on the tendency of an aromatic system to equalize its internal bond lengths. The most popular of these protocols, the harmonic oscillator model of aromaticity (HOMA) [19,20,21], evaluates deviations of bond lengths from a model system, usually taken as benzene, but there are variants of this idea in the literature [21,22,23,24]. There are also various measures of aromaticity that more directly involve energetics in one way or another. But there is no agreement as to what ought to be the standard reference system to evaluate the so-called aromatic stabilization energy [3,25,26,27,28].

But there is a central question that remains unresolved. Are all of these different views of aromaticity consistent with one another? For instance, what does each prescription have to say about the effects of adding a substituent to a benzene ring? It is to this last question that the current work is devoted. Beginning with benzene as a starting point, a host of different substituents are added, both electron-withdrawing and -releasing. Unlike most previous work in this direction that confined the analysis to a single sort of aromaticity measure, this work casts a wider net. In one direction, the response of the system to an external magnetic field is assessed, not just one particular measure but several, as described below. The ability of aromaticity to stabilize the system is rather like a newly caught fish: hard to grasp firmly. Several means of doing so are thus defined and applied here. As mentioned above, a central tenet of an aromatic ring is the equalization of the bond lengths and strengths. Several measures are, therefore, developed here and applied to the various substituted benzenes.

It would be quite desirable if all of the different quantitative evaluations of aromaticity were highly consistent with one another and led to similar conclusions, and to an unambiguous answer as to the effect of each substitution. But, as described in some detail below, such is not the case. The various classes of aromaticity assessments lead, at times, to different substituent effects, pointing to the conclusion that aromaticity is a multifaceted phenomenon, and that each different measure assesses a different aspect.

## 2. Results

The various measures of aromaticity can be grouped into one of three broad categories. The first type relates to the ability of an external field to induce a ring current within the π-cloud of an aromatic system that opposes this field. A second group is based on the propensity of an aromatic system to equalize the properties of the six C-C bonds, as opposed to the alternation of a non-conjugated system. Various energetic manifestations of aromaticity comprise the third category. Each section below defines several measures of a given type, and explores how they are affected by substitutions on the benzene ring.

### 2.1. Magnetic Properties

Several of the most widely used magnetic assessments of aromaticity focus on the response to an externally applied magnetic field by the circulation of the electrons within the π-system. The NICS protocol quantifies this response as the shielding along an axis perpendicular to the molecular plane, that passes through its center. NICS(0) considers the shielding of a point that lies within the plane, while another point 1 Å above the plane NICS(1) is thought to better capture the response of the π-system with less influence of the σ-electrons. A variant of this idea focuses on the component of the shielding that lies parallel to the external magnetic field in the z-direction and is commonly referred to as NICS(0)_zz_ and NICS(1)_zz_, with a similar meaning for the number in parentheses. (While NICS formally defines these values as the negative of the shielding, their actual values are reported here without a change of sign).

The values extracted for these four parameters for each of the substituted benzenes are displayed in Table 1. They are ordered in diminishing value of NICS(1)_zz_, which is largest for the unsubstituted benzene at 30.99 ppm. There is no clear connection between NICS(1)_zz_ and the electron-withdrawing capability of the substituent. For example, the strongly electron-releasing Li is just below H, while NH_2_ offers the lowest value of all. The strongly electron-withdrawing CN and NO_2_ lie roughly in the middle of the pack rather than on either extremity. There is some consistency within the halogen subset, as NICS(1)_zz_ diminishes in the same order as the electronegativity of F > Cl > Br > I. Moving the point of measurement into the plane of the ring cuts the z-component of the shielding roughly in half. Perhaps more to the point, there is also a change in pattern that occurs. It is F that has the highest value of NICS(0)_zz_, larger than H. NH_2_ retains its minimum position, and the halogen ordering remains the same. Li remains near the top but CHO is moved down near the bottom as does COOH.

Rather than focusing on the z-component, the full isotropic shielding parameter offers yet another view on aromaticity. NICS(1) seems to align more closely with ideas concerning electron withdrawal. NO_2_ lies atop the list, followed by F, CN, and COOH. The electron-releasing NH_2_ and OH have some of the lowest values, and one again sees the F > Cl > Br > I ordering. Again, the displacement of the reference point into the molecular plane reduces the field response. The electron-withdrawing F and NO_2_ sit atop the NICS(0) listing, but they are closely followed by OH and OMe which are considered electron-releasing. Moreover, the electron-withdrawing COOH now lies in the middle of the pack of NICS(0), sandwiched between NH_2_ and H.

**Figure 1:**
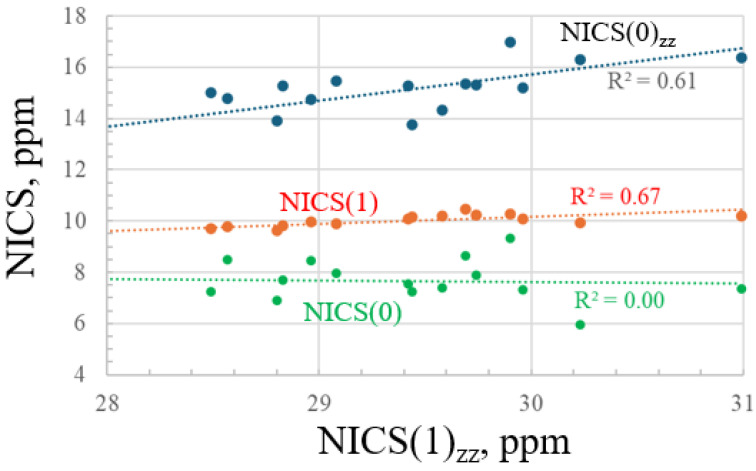
Relationship between various NICS parameters of substituted benzenes.

A graphical summary of possible relationships between the various magnetic parameters is displayed in Figure 1 where NICS(1)_zz_ serves as the abscissa. This parameter is only modestly correlated with NICS(1) and NICS(0)_zz_ with correlation coefficients of 0.67 and 0.61, respectively. There is essentially no relationship between NICS(1)_zz_ and NICS(0). In summary, there are only vague patterns that are consistently followed by various magnetic indicators. All conclude that the halogen effects diminish with the increasing size of the X atom. But there are few other trends that are common to each of these four parameters.

### 2.2. Bond Length Uniformity

Another defining feature of an aromatic ring such as benzene is the equalization of bond lengths, as opposed to the alternating single and double bonds that would occur in its absence. HOMA quantifies the level of bond length equalization within a ring, by evaluating the square of the deviation of each bond length from a standard, which is usually taken as that of benzene. In the same spirit, but recognizing that the inclusion of a substituent would modify all of the bond lengths so that unsubstituted benzene is perhaps not the best reference, this property is evaluated here as the square root of the sum of the square of the deviation of each bond length from the mean for each given substituent:

σ(R) = 1000 × sqrt{Σ(R_i_ − R_avg_)^2^} (1)

where R_avg_ refers to the mean CC length and R_i_ to each individual length. These aromaticity measures are listed in the first column of Table 2. One can apply the same philosophy, not to the bond lengths, but rather to their strengths [29], as measured by the density at the AIM bond critical point.

σ(ρ) = 1000 × sqrt{Σ(ρ_i_ − ρ_avg_)^2^} (2)

These values of σ(ρ_BCP_) are presented in the next column of Table 2. Lastly, the AIM analysis of a ring structure such as the substituted benzenes will normally contain a ring critical point (RCP) at its center. The electron density at this point may have some bearing on the level of aromaticity, and so is reported in the last column of Table 2.

The perfect symmetry of the unsubstituted benzene leads to σ(R) values of zero, which can be considered as full aromaticity. Halogen substituents reduce this aromaticity, with I being the smallest perturbation and F the largest. The electron-releasing NH_2_, OMe, and Li groups yield the largest perturbations, but this effect is not solely due to electronegativity as the effect of CN is nearly as large, and NO_2_ is not far behind. The pattern for σ(ρ_BCP_) is similar in certain respects but distinctly different in others. The I, again, perturbs the symmetry least of the halogens and F the most. Li repeats as the most perturbing substituent. On the other hand, OH is more perturbing to the bond densities than to the bond lengths, whereas Me and NH_2_ behave in the opposite manner.

The ring critical point densities in the last column are all very similar, varying over a narrow range from 0.0241 to 0.0248, so one is reluctant to draw many conclusions here. Indeed, the largest values occur with NO_2_ and Li which are, respectively, the most electron-withdrawing and -releasing groups. There does appear to be a tendency for the electron-releasing NH_2_, OH, and OMe groups to have the smallest ρ_RCP_, but, again, the small differences from one group to the next argue against drawing conclusions from this fact.

**Figure 2:**
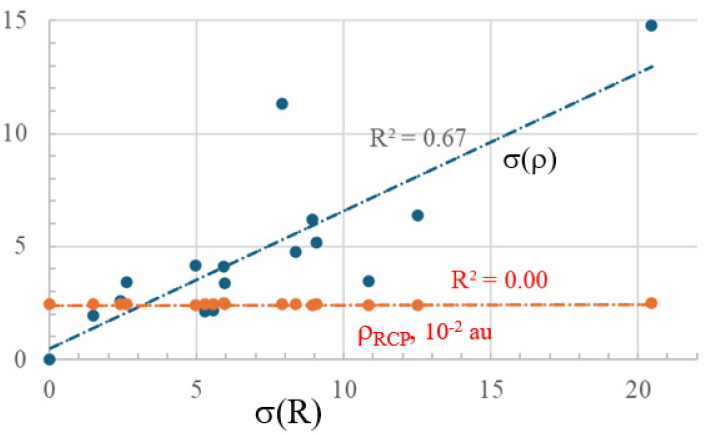
Relationship between deviations from uniformity of internal C-C bond lengths, and that between their AIM bond critical point densities, and the density of the ring critical point.

The relationship between the σ(R) based on bond lengths, and the equivalent parameter extracted from bond critical point densities is visible as the blue curve in Figure 2. The correlation coefficient of 0.67 is only modest so these two quantities exhibit certain differences from one another. The red line pertaining to the ring critical point density is essentially flat, with little dependence upon the nature of the substituent. Therefore, a HOMA-like analysis of bond length alternation provides a different view of aromaticity than does an analogous view derived from bond critical point densities, all of which are, again, distinct from parameters drawn from the response of the ring to an external magnetic field.

### 2.3. Energetic Measures

**Figure 3:**
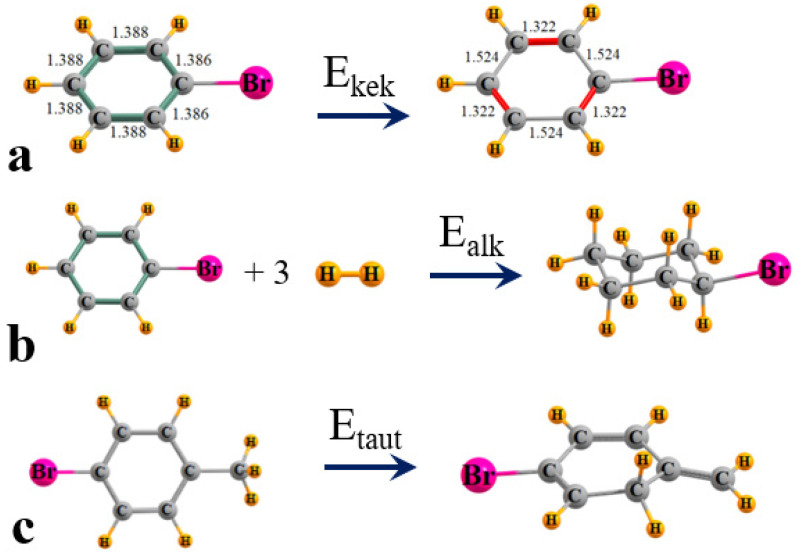
Definition of energetic measures of aromaticity for bromobenzene as an example.

While it is widely recognized that aromaticity stabilizes a system, it is difficult to pinpoint precisely what that means, that is, what might be a reasonable specific definition of this stabilization energy. A review of this topic [3] acknowledged the ambiguity of defining such an energetic measure, and described a diversity of attempts in this direction, as, for example, the protonation energy or heat of hydrogenation [30]. Figure 3a proposes one definition which compares the energy of the fully optimized aromatic system on the left with a variant on the right which comprises one of the two Kekule structures. Specifically, each geometry was fully optimized but with the restriction of alternating single and double bonds, whose lengths were derived from optimizations of ethane and ethylene, respectively. For purposes of emphasis, the longer single bonds are rendered in grey while red indicates the shorter double bonds.

The difference in energy between these two geometries is defined as E_kek_, which is listed in the first column of Table 3 for the various substituted benzenes. These energy differences span the range between 16.52 kcal/mol for the Li derivative, up to 19.23 kcal/mol for the n-propyl group. There are no obvious trends in the data. For example, E_kek_ drops down by more than 2 kcal/mol when the n-propyl group is simply shortened to its methyl analogue, which lies near the bottom of the list. E_kek_ is much larger for OMe than for OH. On the other hand, the size of the substituent alters in the opposite direction for the halogens with F > Cl > Br > I. Strongly electron-withdrawing groups like F, NO_2_, and COOH are near the top of the list, along with the electron-releasing OMe. CN and NH_2_, near opposites in this regard, have identical values of E_kek_.

An alternate energetic definition is summarized in Figure 3b and relates the difference in energy between the aromatic system on the left, and a fully aliphatic substituted cyclohexane on the right. Since a direct comparison is not feasible as there are six additional H atoms in the aliphatic system, and the equation is unbalanced, these atoms are added to the left side in the form of three H_2_ molecules. The energetics of this reaction in Figure 3b which converts the aromatic to the corresponding alkane system is designated as E_alk_. One may consider that a larger amount needed to raise the energy of the aromatic to an aliphatic might be one energetic measure of aromatic stability.

As may be seen in the next column of Table 3, E_alk_ follows a largely different pattern than does E_kek_. The former is largest for the halogen substituents, and in the reverse order: I > Br > Cl > F, as most of the preceding measures of aromaticity. The electron-withdrawing NO_2_ and CN groups are near the top of the list, and COOH only slightly below. Electron-releasing groups tend toward lower E_alk_, with Li at the bottom, and NH_2_, CHO, Ph, and OH rising in that order.

Yet a third and different means of assessing the energetic consequence of aromaticity also arises from the disruption of the planarity of the aromatic system, along with its alternating conjugated set of three double bonds [31]. This disruption is caused by first placing a CH_3_ group para to the substituent of interest as in the left side of Figure 3c for Br. The forced migration of one of the three methyl H atoms to the neighboring C on the ring leads to the tautomer on the right side with methyl now being replaced by a =CH_2_ group, and there are no double bonds to the CH_2_ within the ring with its sp^3^ hybridization. The deviation from the planarity is now clearly evident, another factor in the deletion of aromaticity.

The energy of this H migration and tautomerization is referred to here as E_taut_, and is listed in the next column of Table 3. E_taut_ is equal to 34.1 kcal/mol for unsubstituted benzene, and spans a range of about 4 kcal/mol, between 32.5 and 36.3 kcal/mol. It does not appear to scale with the electron-withdrawing or -donating capacity of the substituent. For example, E_taut_ reaches its minimum for the electron-donating n-propyl group, and its maximum is realized for another donating group, OMe. The highly potent withdrawing NO_2_ substituent yields very little change to benzene itself.

The energy gap between the HOMO and LUMO is often taken as one measure of certain aspects of a molecule’s reactivity or its conductivity properties. This gap is contained in the next column of Table 3 and displays another pattern than do the other quantities. For example, NO_2_ and COOH are both highly electron-withdrawing but they have quite different values of E_gap_, with the electron-releasing NH_2_ lying between them. Unsubstituted benzene contains the largest gap, which conforms to the idea that benzene is the prototype aromatic ring. On the other hand, there is again a clear pattern within the halogen subset F > Cl > Br > I.

**Figure 4:**
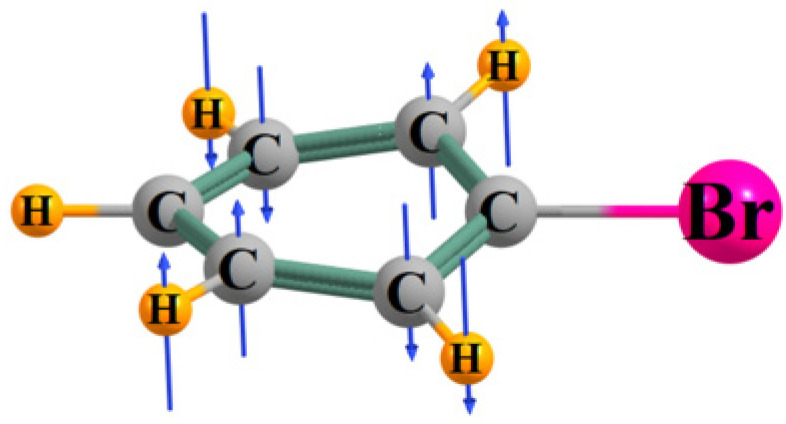
Motions of atoms involved in the out-of-plane normal vibrational mode of the aromatic ring.

Another analytical measure of the pull toward planarity can be expressed by the frequency of the normal mode vibration that moves the atoms in a sort of nonplanar motion. The particular mode of interest is diagrammed in Figure 4 where the blue arrows indicate the direction and amplitude of motion within this mode. The Br, the C to which the Br substituent is attached, and the para-CH group are nearly stationary so this motion ought to be minimally affected by the mass of each substituent. The mode draws the two CH groups ortho to Br in opposite directions, and the same is true for the two meta CH groups, so it is a true nonplanar motion.

The frequency of this mode contained in the last column of Table 3 spans a range between 388 cm^−1^ for Li up to a maximum of 426 cm^−1^ for OMe. Some of the groups with the highest frequency are electron-releasing such as OMe, OH, and NH_2_, but the electron-withdrawing F, NO_2_, and Cl are also associated with high frequencies. The halogens maintain their usual F > Cl > Br > I order.

**Figure 5:**
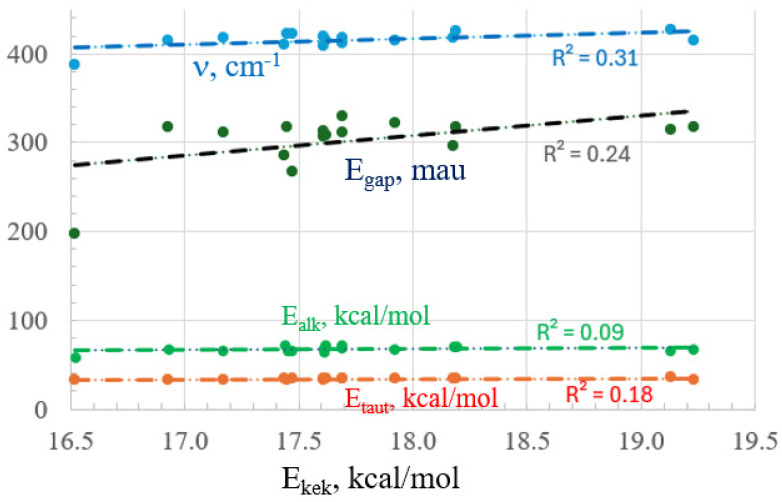
Relationship of E_kek_ with E_alk_, E_taut_, HOMO-LUMO gap, and ν.

Overall, these five independent energetic measures of aromaticity offer quite different assessments as to the effects of substituents. The patterns have little in common with one another, nor with physical organic estimates of electron-withdrawing strength. The relationships are illustrated graphically in Figure 5, where the very low correlation coefficients emphasize the different patterns of these various energetic measures of aromaticity. The relationship between E_alk_ and ν is also poor, with a correlation coefficient of 0.19.

There have been several earlier attempts to correlate certain aspects of aromaticity in substituted benzenes and related systems. Krygowski [19] had concluded that the equalization of bond lengths in the HOMA context within p-NO_2_ benzenes depends on both σ and π-electron effects. Krygowski et al. [25] considered several options to compute an aromatic stabilization energy but none seemed to correlate well with the Hammet constants, nor were several NICS indices excellent in this regard. A more recent effort [32] applied variants of NICS, assessed ring currents, and the electron density of delocalized bonds (EDDB). These authors noted that electron-withdrawing/releasing substituents raised/lowered ring currents. Given certain inconsistencies with NICS(1)_zz_ and NICS(0)_zz_, they concluded that no single method can be reliably depended upon. Several reviews [3] had acknowledged common disagreements between geometrical, energetic, and magnetic measures of aromaticity, agreeing with the recommendation made here that several of these criteria should be considered in each case. These authors had summarized the idea [6] that, rather than considering the evaluation of the NICS index at one or two selected points, a wide range of points dispersed over space might offer another perspective, including perhaps an integration of these computed values over some region of space [33,34].

**Figure 6:**
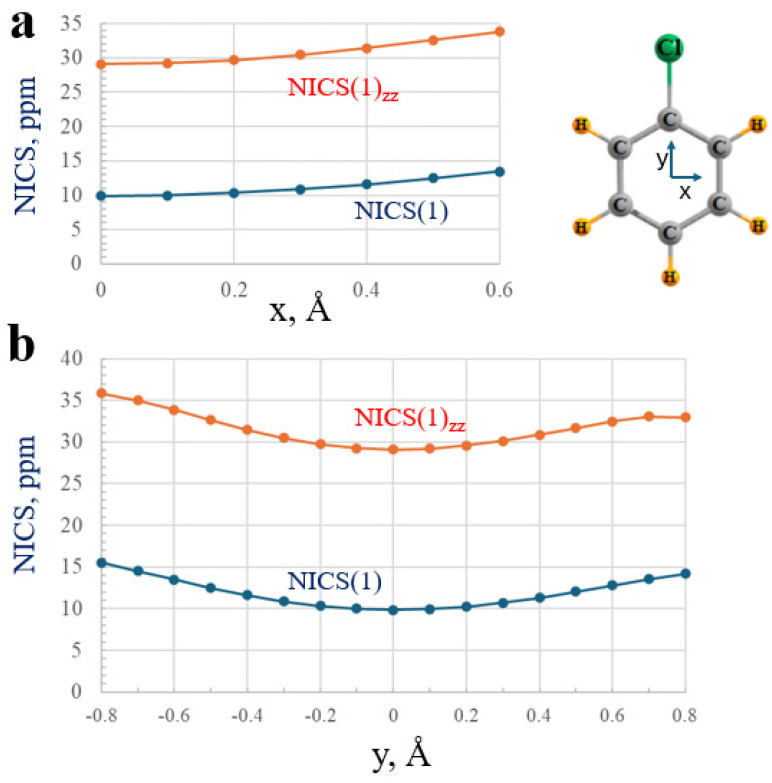
Variation of NICS parameters as the point of reference is moved in the (**a**) x- and (**b**) y-directions from the center within a plane located 1 Å above the plane of chlorobenzene.

As an elaboration of this idea, it is generally presumed that one should measure the NICS indices along an axis perpendicular to the molecular plane, and passing through the center of the ring, as was performed above. As an exploration of this idea, NICS quantities were evaluated within a plane lying 1 Å above the plane of chlorobenzene, but at varying distances from the point lying directly over the ring center. As depicted in Figure 6, both NICS(1) and NICS(1)_zz_ grow larger as one moves in either the x- or y-direction away from this center. As an example of the quantitative variation, a 0.6 Å motion in the x-direction raises NICS(1) and NICS(1)_zz_ by 36% and 16%, respectively. The use of a small inert atom like He as a probe of the magnetic shielding offers a very different set of NICS parameters than does a dummy atom bearing no electrons. For example, NICS(1) and NICS(1)_zz_ are substantially larger with He as the probe: NICS(1) grows by a factor of 7 and NICS(1)_zz_ is tripled.

It might be noted, finally, that the effects of the substituents on the aromaticity properties of benzene have been rather small. Recent work [35] observed much larger effects on the first excited triplet electronic state, which might perhaps have important implications for certain sorts of chemical reactivity. Indeed, there is a burgeoning field dealing with how aromaticity can affect the transition states and, thereby, reactivity in various processes such as the Diels–Alder reaction [36].

## 3. Methods

Quantum chemical calculations were performed via the density functional approach (DFT), within the context of the M06-2X functional [37]. A polarized triple-ζ def2-TZVP basis set was chosen so as to afford a large and flexible set. Geometries were fully optimized and verified as true minima by the absence of imaginary vibrational frequencies. The Gaussian 16 [38] program was chosen as the specific means to conduct these computations. Atoms in molecules (AIM) bond paths and their associated critical points [39] were located and their properties evaluated with the aid of the Multiwfn program [40].

## 4. Conclusions

While all methods applied here are, of course, consistent in that the substituted benzenes are all aromatic, there is little agreement as to which substituents might enhance or diminish this property. Within the context of the NICS response to an external magnetic field, the trends differ depending upon whether the point considered lies within the ring or above it, and changes when the perpendicular component is considered versus its isotropic average. Deviations of the properties of the individual C-C bonds from their mean suggest different substituent effects, whether it is the bond length or the bond critical point density that is considered. There is also a discrepancy within the energetic measures of aromaticity, whether in a comparison of the system with its Kekule structure with alternating single and double C-C bonds, the reaction energy that mutates the molecule to an aliphatic cyclohexyl analogue, the energy required to place an extra H on the ring and, thereby, disturbing its conjugation, the HOMO-LUMO gap, or the vibrational frequency of the mode that pulls the system out of its equilibrium planar arrangement. While all of these measures of aromaticity are valid in and of themselves, one should exercise due caution in drawing conclusions from any one approach.

The diversity of the results here reinforces the notion that the aromaticity phenomenon is a multiheaded beast, and so is not fully gauged by any single property. The situation puts one in mind of the fable of several blind men each feeling a different part of an elephant. It is perhaps naive to think that any single measure could fully capture the full range of what is commonly attributed to aromaticity: all aspects of the response to a magnetic field, uniformity of various properties of the bonds, and a host of energetic measures. The comparison of the aromaticity of several related molecules by any single measure can certainly be deemed valid, but it must be understood that it only captures one of the many facets of aromaticity. Therefore, a more complete understanding is predicated on the application of several measures, each of which presents its own partial picture of this complex phenomenon.

## Figures and Tables

**Table 1:** Magnetic field response aspects of aromaticity of substituted benzenes, all in ppm.

	NICS(1)_zz_	NICS(0)_zz_	NICS(1)	NICS(0)
H	30.99	16.37	10.21	7.35
Li	30.23	16.30	9.92	5.97
Me	29.96	15.19	10.07	7.33
F	29.90	16.98	10.27	9.31
CN	29.74	15.33	10.24	7.88
NO_2_	29.69	15.36	10.46	8.64
COOH	29.58	14.34	10.18	7.38
CHO	29.44	13.76	10.17	7.23
n-propyl	29.42	15.27	10.07	7.53
Cl	29.08	15.46	9.88	7.95
OMe	28.96	14.73	9.97	8.45
Br	28.83	15.28	9.82	7.70
Ph	28.80	13.91	9.62	6.91
OH	28.57	14.78	9.77	8.49
I	28.49	14.99	9.70	7.25
NH_2_	27.06	12.02	9.23	7.42

**Table 2:** Deviations from average of CC bond lengths R and CC bond critical point density, and density of ring critical point.

	σ(R)	σ(ρ_BCP_)	100× ρ_RCP_
H	0.00	0.00	2.44
I	1.48	1.93	2.46
Br	2.40	2.59	2.45
Cl	2.62	3.44	2.44
OH	4.95	4.15	2.41
n-propyl	5.29	2.13	2.44
Me	5.55	2.16	2.44
NO_2_	5.90	4.11	2.47
COOH	5.96	3.38	2.45
F	7.91	11.31	2.43
CHO	8.35	4.74	2.46
CN	8.92	6.18	2.42
Ph	9.07	5.19	2.44
NH_2_	10.84	3.47	2.41
OMe	12.51	6.38	2.41
Li	20.46	14.77	2.48

**Table 3:** Energetic quantities (defined in Figure 3, kcal/mol), HOMO-LUMO gap (10^−3^ au), and out-of-plane bending frequency (cm^−1^) of mode exhibited in Figure 4.

	E_kek_	E_alk_	E_taut_	E_gap_	ν
n-propyl	19.23	67.04	32.54	317	415.47
OMe	19.13	65.96	36.30	315	426.41
F	18.19	70.58	33.88	318	425.44
NO_2_	18.18	70.95	34.07	296	418.61
COOH	17.92	67.04	34.35	322	414.88
Cl	17.69	71.23	33.81	312	417.87
H	17.69	68.25	34.11	329	412.06
Br	17.62	72.11	33.76	308	414.25
CN	17.61	69.06	34.20	307	409.10
NH_2_	17.61	63.86	33.66	313	418.85
Ph	17.47	65.69	34.17	267	422.28
OH	17.45	65.95	33.25	317	422.26
I	17.44	72.17	33.73	285	409.91
CHO	17.17	65.68	32.78	311	417.35
Me	16.93	67.88	33.04	317	414.98
Li	16.52	58.25	33.18	198	387.88

## Data Availability

Original data available upon request to authors.

## References

[1] Hofmann A.W.I. (1857). On insolinic acid. Proc. R. Soc. Lond..

[2] Krygowski T.M., Cyrañski M.K., Czarnocki Z., Häfelinger G., Katritzky A.R. (2000). Aromaticity: A Theoretical Concept of Immense Practical Importance. Tetrahedron.

[3] Chen Z., Wannere C.S., Corminboeuf C., Puchta R., Schleyer P.V. (2005). Nucleus-Independent Chemical Shifts (NICS) as an Aromaticity Criterion. Chem. Rev..

[4] Feixas F., Matito E., Poater J., Solà M. (2015). Quantifying aromaticity with electron delocalisation measures. Chem. Soc. Rev..

[5] Baranac-Stojanović M. (2017). 4π-Electron B–N Monocycles: Stability and (Anti)aromaticity. Eur. J. Org. Chem..

[6] Stanger A. (2010). Obtaining Relative Induced Ring Currents Quantitatively from NICS. J. Org. Chem..

[7] Stanger A. (2009). What is… aromaticity: A critique of the concept of aromaticity—Can it really be defined?. Chem. Commun..

[8] Szatylowicz H., Jezuita A., Krygowski T.M. (2019). On the relations between aromaticity and substituent effect. Struct. Chem..

[9] Katritzky A.R., Barczynski P., Musumarra G., Pisano D., Szafran M. (1989). Aromaticity as a quantitative concept. 1. A statistical demonstration of the orthogonality of classical and magnetic aromaticity in five- and six-membered heterocycles. J. Am. Chem. Soc..

[10] Jiao H., von Ragué Schleyer P. (1995). The Cope Rearrangement Transition Structure Is Not Diradicaloid, but Is It Aromatic?. Angew. Chem. Int. Ed. Engl..

[11] Cysewski P., Jelinski T., Krygowski M.T., Oziminski P.W. (2012). Factors Influencing Aromaticity: PCA Studies of Monosubstituted Derivatives of Pentafulvene, Benzene and Heptafulvene. Curr. Org. Chem..

[12] Gershoni-Poranne R., Stanger A. (2015). Magnetic criteria of aromaticity. Chem. Soc. Rev..

[13] Gomes J.A.N.F., Mallion R.B. (2001). Aromaticity and Ring Currents. Chem. Rev..

[14] Leyva-Parra L., Pino-Rios R., Inostroza D., Solà M., Alonso M., Tiznado W. (2024). Aromaticity and Magnetic Behavior in Benzenoids: Unraveling Ring Current Combinations. Chem. Eur. J..

[15] Schleyer P.v.R., Maerker C., Dransfeld A., Jiao H., van Eikema Hommes N.J. (1996). Nucleus-Independent Chemical Shifts:  A Simple and Efficient Aromaticity Probe. J. Am. Chem. Soc..

[16] Sundholm D., Fliegl H., Berger R.J.F. (2016). Calculations of magnetically induced current densities: Theory and applications. WIREs Comput. Mol. Sci..

[17] Geuenich D., Hess K., Köhler F., Herges R. (2005). Anisotropy of the Induced Current Density (ACID), a General Method to Quantify and Visualize Electronic Delocalization. Chem. Rev..

[18] Herges R., Geuenich D. (2001). Delocalization of Electrons in Molecules. J. Phys. Chem. A.

[19] Krygowski T.M. (1993). Crystallographic studies of inter- and intramolecular interactions reflected in aromatic character of .pi.-electron systems. J. Chem. Inf. Comp. Sci..

[20] Kruszewski J., Krygowski T.M. (1972). Definition of aromaticity basing on the harmonic oscillator model. Tetrahedron Lett..

[21] Mischie A., Toader A.M., Buta M.C., Cimpoesu F. (2023). Harmonic oscillator model of aromaticity (HOMA) in conjugated radicals and cations. Comput. Theor. Chem..

[22] Stawski W., Zhu Y., Wei Z., Petrukhina M.A., Anderson H.L. (2023). Crystallographic evidence for global aromaticity in the di-anion and tetra-anion of a cyclophane hydrocarbon. Chem. Sci..

[23] Arpa E.M., Durbeej B. (2023). HOMER: A reparameterization of the harmonic oscillator model of aromaticity (HOMA) for excited states. Phys. Chem. Chem. Phys..

[24] Krygowski T.M., Cyrański M. (1996). Separation of the energetic and geometric contributions to the aromaticity. Part IV. A general model for the π-electron systems. Tetrahedron.

[25] Krygowski T.M., Ejsmont K., Stepień B.T., Cyrański M.K., Poater J., Solà M. (2004). Relation between the Substituent Effect and Aromaticity. J. Org. Chem..

[26] Herndon W.C., Mills N.S. (2005). Aromatic Stabilization Energy Calculations for the Antiaromatic Fluorenyl Cation. Issues in the Choice of Reference Systems for Positively Charged Species. J. Org. Chem..

[27] Cyrański M.K. (2005). Energetic Aspects of Cyclic Pi-Electron Delocalization:  Evaluation of the Methods of Estimating Aromatic Stabilization Energies. Chem. Rev..

[28] Aihara J.-i. (2016). Graph Theory of Aromatic Stabilization. Bull. Chem. Soc. Jpn..

[29] Kemula W., Krygowski T.M. (1968). Substituent effects in poorly aromatic system: Dichloro-anthraquinones and related semi-antrhraquinone radicals. Tetrahedron Lett..

[30] Stanger A. (2008). The Different Aromatic Characters of Some Localized Benzene Derivatives. J. Phys. Chem. A.

[31] Schleyer P.v.R., Pühlhofer F. (2002). Recommendations for the Evaluation of Aromatic Stabilization Energies. Org. Lett..

[32] Patra S.G., Mondal H., Bhattacharjya M.J., Chetia N., Chattaraj P.K. (2023). On the aromaticity of substituted benzene. Theor. Chem. Acc..

[33] Stanger A. (2019). Reexamination of NICSπ,zz: Height Dependence, Off-Center Values, and Integration. J. Phys. Chem. A.

[34] Stanger A. (2020). NICS—Past and Present. Eur. J. Org. Chem..

[35] Baranac-Stojanović M. (2020). Substituent Effect on Triplet State Aromaticity of Benzene. J. Org. Chem..

[36] Zhu Q., Chen S., Chen D., Lin L., Xiao K., Zhao L., Solà M., Zhu J. (2023). The application of aromaticity and antiaromaticity to reaction mechanisms. Fundam. Res..

[37] Zhao Y., Truhlar D.G. (2008). The M06 suite of density functionals for main group thermochemistry, thermochemical kinetics, noncovalent interactions, excited states, and transition elements: Two new functionals and systematic testing of four M06-class functionals and 12 other functionals. Theor. Chem. Acc..

[38] Frisch M.J., Trucks G.W., Schlegel H.B., Scuseria G.E., Robb M.A., Cheeseman J.R., Scalmani G., Barone V., Petersson G.A., Nakatsuji H. (2016). Gaussian 16 Rev. C.01.

[39] Bader R.F.W. (1990). Atoms in Molecules, A Quantum Theory.

[40] Lu T., Chen F. (2012). Multiwfn: A multifunctional wavefunction analyzer. J. Comput. Chem..

